# Composite Anion Exchange Membranes Fabricated by Coating and UV Crosslinking of Low-Cost Precursors Tested in a Redox Flow Battery

**DOI:** 10.3390/polym13152396

**Published:** 2021-07-21

**Authors:** Martyna Charyton, Francesco Deboli, Peter Fischer, Gerard Henrion, Mathieu Etienne, Mateusz L. Donten

**Affiliations:** 1 Amer-Sil S.A., 61 Rue D’Olm, Kehlen, 8281 Luxembourg, Luxembourg; martyna.charyton@amer-sil.com (M.C.); francesco.deboli@amer-sil.com (F.D.); 2 CNRS, Institut Jean Lamour (IJL), Université de Lorraine, 2 Allée André Guinier, F-54011 Nancy, France; gerard.henrion@univ-lorraine.fr; 3 CNRS, Laboratory of Physical Chemistry and Microbiology for the Environment (LCPME), Université de Lorraine, 405 Rue de Vandoeuvre, F-54600 Villers-lès-Nancy, France; mathieu.etienne@univ-lorraine.fr; 4 Department of Chemical Engineering, KU Leuven, Celestijnenlaan 200F, B-3001 Leuven, Belgium; 5 Electrochemistry, Fraunhofer Institute for Chemical Technology ICT, Joseph-von-Fraunhofer, Straße 7, 76327 Pfinztal, Germany; peter.fischer@ict.fraunhofer.de

**Keywords:** composite ion exchange membrane, functional coating, UV curing, poly(vinyl pyrrolidone), redox flow battery

## Abstract

This paper presents a novel, cost-effective approach to the fabrication of composite anion exchange membranes (AEMs)**.** Hierarchical AEMs have been fabricated by coating a porous substrate with an interpenetrating polymer network (IPN) layer where poly(vinylpyrrolidone) (PVP) is immobilized in a crosslinked matrix. The IPN matrix was formed by UV initiated radical crosslinking of a mixture of acrylamide-based monomers and acrylic resins. The fabricated membranes have been compared with a commercial material (Fumatech FAP 450) in terms of ionic transport properties and performance in a vanadium redox flow battery (VRFB). Measures of area-specific resistance (ASR) and vanadium permeability for the proposed membranes demonstrated properties approaching the commercial benchmark. These properties could be tuned by changing the content of PVP in the IPN coating. Higher PVP/matrix ratios facilitate a higher water uptake of the coating layer and thus lower ASR (as low as 0.58 Ω.cm^2^). On the contrary, lower PVP/matrix ratios allow to reduce the water uptake of the coating and hence decrease the vanadium permeability at the cost of a higher ASR (as high as 1.99 Ω.cm^2^). In VRFB testing the hierarchical membranes enabled to reach energy efficiency comparable with the commercial AEM (PVP_14—74.7%, FAP 450—72.7% at 80 mA.cm^−2^).

## 1. Introduction

Electromembrane processes are at the core of many modern technologies for energy storage and conversion, water treatment and resource recovery such as redox flow batteries, fuel cells and electro-dialyzers [1,2,3]. Ion exchange membranes (IEMs) are materials essential to all of them and strongly affect their performance and economic viability. As such, they can be seen critical for addressing energy management and resource scarcity, which are some of the most pressing issues in the modern society [4]. New cost-effective methods of producing such membranes are an important enabler for further widespread of redox flow batteries and other electro membrane process dependent technologies. Hierarchical IEMs fabricated by coating a porous substrate with an ion exchange layer allow to include low cost and environmentally advantageous production processes as well as ionomer chemistries not strongly present in the current market of IEMs. This work aims at applying the expertise of the coating and UV cure industries for fabrication of IEMs.

Ion exchange membranes are typically thin films designed to selectively permeate targeted ions, while blocking others by charge exclusion or affinity driven mechanisms [5,6]. Fundamentally, the selectivity of this type of membrane is achieved through electrostatic interactions between the charge of fixed ionic groups present in the material and the migrating ion [7]. Membranes having negatively charged fixed groups will permeate cations, while membranes having positively charged groups will permeate anions [4,8,9]. The applicability of a given membrane is strongly determined by its ionic conductivity in combination with ion selectivity and its chemical stability under the process conditions. Ionic transport properties are often put forward in the consideration since in all electromembrane processes, ion exchange membranes are expected to minimize energy losses or, in other words, allow to reach high efficiencies. In parallel, many of the applications of such membranes require their operation in environments of extreme pH as well as in oxidative conditions [4,10,11].

Energy storage technologies such as redox flow batteries (RFBs) can be given as an example to illustrate all of these performance and material requirements. In RFB cells the membrane separates the positive and negative electrolytes between which the electric potential is generated [12]. The role of the membrane is to block the transfer of redox-active species (ionic) between the two electrolytes. Such transfer would lead to self-discharge of the RFB which results in a capacity loss [13]. Conduction of the common ion across the membrane is needed to close the electric circuit of the battery and balance the ongoing electrode reactions [14]. Therefore, the membrane’s ohmic resistivity must be minimized to diminish the energy losses [5]. Finally, most RFBs operate under strongly acidic conditions and by their nature contain highly oxidative compounds such as metal ions at high oxidation states (vanadium (V), iron (III) or cerium (IV)), bromine, or highly reactive-oxidative organic molecules to name a few used in the various RFB chemistries studied to date [15,16,17,18,19,20,21]. The membrane is expected to withstand years-long operation under such conditions typical for the life-time of battery systems [10].

Ion selective, yet well ion conductive membranes are often fabricated out of multiblock ionic copolymers having a complex architecture of hydrophobic and hydrophilic domains [12]. The hydrophobic domains provide the material with mechanical integrity and dimensional stability limiting the ionomer swelling and allowing it to form a self-standing foil. The hydrophilic domains, containing the charged groups, substantiate the ion conducting channels and determine the membrane’s charge selectivity [22]. The overall ionic conductivity and selectivity of the membrane can be controlled by designing the combination and ratio of the two types of domains within the range in which the film remains intact in the applied conditions [23].

Perfluorinated sulfonic acid (PFSA)-based cation exchange membranes such as Nafion^®^ [24] are built of domains as described above. Historically these membranes were designed as proton exchangers in the harsh environment of fuel cells, where the chemical stability of the fluorinated backbone represents a considerable advantage [25]. Nafion membranes were successfully applied in RFB (all vanadium) showing promising performance and excellent stability in the highly oxidative media [26,27]. However, the use of PFSA as the main building block predetermines the properties of the membrane and results in a relatively high final cost of the membrane caused by chemical synthesis of the perfluorinated polymer and non-trivial processing into thin films [28].

The emergence of applications with milder process conditions, such as organic redox flow batteries, has led to the development of membranes based on a non-fluorinated polymer backbone [29]. This evolution was driven by the aim to reduce the cost and the environmental impact of such materials [9]. Sulfonated or quaternary ammonium poly(arylene ether ketones) (PAEK), polysulfones (PSU), polyphenylene oxides (PPO) or polyimides (PI) have been reported as promising ion exchange polymers for these applications [30,31,32,33,34]. Crosslinked ionomers have also been investigated for the application in ion exchange membranes [35] as the degree of crosslinking can be used to tune membrane properties [36]. Densely crosslinked ionic polymers exhibit better dimensional stability and reach good mechanical toughness [37]. They are likely to achieve enhanced selectivity at the cost of a lower ionic conductivity, due to the limited space between the polymeric chains. In a similar way, loosely crosslinked ionomers will show an improved ion conductivity but lower dimensional stability and selectivity [38].

All of the above membrane materials, regardless of their details, usually rely on a single copolymer for achieving both mechanical and ionic transport properties of the membrane [4,39]. Their success requires precise balancing of the various domains of the polymer, which implies non-trivial synthesis and complex fabrication processes. Both of these aspects contribute to a high cost of the final material. This problem is often pointed out as a factor slowing down the widespread utilization of electromembrane based technologies in the energy and resource recovery domains [40,41].

Composite membranes can be considered an interesting alternative in which simpler materials are used in combination to jointly provide specific properties to the membrane. The key advantage of composite membranes is splitting the requirements for mechanical and transport properties between different components. Ionomers are selected to conduct ions and are embedded or attached to chemically stable and mechanically robust matrices. Some of the most common implementations of composite membranes are made by heterogeneous incorporation of powdered ion exchange resins into polymers such as poly(vinyl chloride) (PVC) or acrylonitrile copolymers [4,6,42,43]. Other types of composite membranes are pore-filled and hierarchical membranes in which the ionomer is placed inside the pores of a substrate or as a layer on top of it [44]. Pore-filled membrane tested in vanadium redox flow batteries demonstrated cycling performance comparable with homogeneous membranes [45]. Hierarchical membranes allow the use of very thin films of functional polymer often not suitable for the fabrication of self-supported films [46]. Composite membranes usually show a lower conductivity and limiting current compared to homogeneous ones, as not all of the components participate in the ionic transport. Despite specific challenges, they find successful applications and offer price advantages owing to relatively low cost of polymers and processing [6].

This paper presents a novel approach to the fabrication of hierarchical ion exchange membranes by blade-coating and UV curing a layer of anion exchange polymer on top of a porous substrate. In light of its surface properties, optimal for the coating process and the subsequent adhesion of the functional layer, a composite PVC–SiO_2_ porous membrane was used as a substrate. Similar porous membranes have been tested as separators for RFBs by other groups, that reported excellent conductivity but only sufficient selectivity [47,48,49]. The ion exchange coatings proposed in this work are expected to improve the latter property, important for the battery performance.

The fabrication process of hierarchical membranes is limited to three steps in which low-cost reagents are formulated, applied on the substrate and rapidly cured under UV light. Contrarily to many common ion exchange membranes, this fabrication method does not require dedicated synthesis or solvent intense processes [8].

The anion exchange coatings were fabricated using a water-soluble polymer–poly (vinyl pyrrolidone) (PVP). PVP is a commodity reagent, widely used as a hydrophilic polymer in the preparation and modification of filtration membranes [50,51] or as a precursor for ion exchange membrane fabrication. In recent works, membrane containing PVP were positively tested for application in VRFBs [52,53]. In highly acidic conditions the pyrrolidone segments can be protonated, facilitating ion conductivity and reducing vanadium permeation due to a positive charge [54]. In this study, PVP was immobilized in a densely crosslinked matrix to create an interpenetrating polymer network (IPN). Radically reactive anion exchange precursors were used to form the IPN matrix in order to contribute to the ion transport properties of the coating layer. The crosslinked matrix allows to control the swelling of PVP while keeping the coating hydrophilic and conductive.

Membranes with different PVP/matrix ratios were fabricated in order to investigate the effect of PVP content on the membrane ion transport properties and performance in vanadium redox flow batteries (VRFBs).

## 2. Materials and Methods

### 2.1. Materials

The reagents used for the fabrication of ion-exchange coating layers and their suppliers’ details are listed in Table 1. PVC-Silica porous substrates with median pore size 60 nm, volume porosity 60.3%, and a thickness of 600 µm were produced internally at Amer-Sil SA, Luxembourg. PVC-Silica membranes are commonly used as separators in lead acid batteries. Due to their excellent wettability and high volume porosity they allow to reach a high ionic conductivity when soaked with battery electrolyte. They also offer advantages when used as a coating substrate since the silica particles exposed at the surface of the membrane provide excellent adhesion of the coating layer due to their wettability and roughness. A SEM micrograph of the surface of the substrate is available in Supplementary Material, Figure S1.

A commercially available homogeneous anion exchange membrane-FAP 450 (Fumatech GmbH, Ingbert, Germany)-was purchased at Fuel Cell Store Inc., USA and used for performance comparison.

### 2.2. Membrane Fabrication

To prepare the ion exchange coating formulation, the liquid precursors DMEA and HEAA were mixed according to the ratio given in Table 2. PVP and MBAAM were then dissolved in this mixture by sonication at moderate temperature (40 °C, 1 h). Subsequently, the viscous acrylate resin–EBE 830–was added and the mixture sonicated at 40 °C for additional 30 min. The photo-initiator I500 was finally added, and the formulation mechanically mixed for 10 min to obtain a homogeneous solution.

The uncured formulation was applied on the porous substrate by means of blade coating using an applicator with a gap of 60 µm and an automatic driver (BYK GmbH, Wesel, Germany). In the final step the coating was cured by radical polymerization (radical curing) under UV irradiation (UV conveyor Jenton International Ltd., Whitchurch, UK) equipped with a 2000 W Fe doped Hg UV lamp (365 nm).

### 2.3. Scanning Electron Microscopy (SEM)

The surface and cross-section of the fabricated membranes were observed under a field emission scanning electron microscope (FESEM Merlin Carl Zeiss Microscopy GmbH, Jena, Germany), coupled with energy dispersive spectroscopy (EDS) (Quantax 400 EDS detector, Bruker, Billerica, MA, USA) in order to assess the ion exchange coating continuity and the nitrogen distribution. The samples were prepared by depositing ca. 1 nm of Au-Pd alloy on their surfaces by plasma sputtering (Quantum SC7620, Quorum Technologies LTD, Laughton, UK).

### 2.4. Water Uptake

Water uptake (*WU*) is commonly used to characterize ion exchange materials. It was measured for the ionomer coating of the hierarchical membranes. For the composite material *WU* of the ionomer needs to be distinguished from water absorption into the pores of the substrate. The objective of the characterization measurement was to identify the impact of the composition of the ionomer coating on its water uptake.

To obtain samples of the coating material free of the porous substrate the coating formulations were casted and cured on a PTFE sheet, then delaminated to obtain flakes. The samples were soaked in deionized water for 24 h to ensure full hydration. After removing the excess water using absorbent paper, the fragile samples were weighted to obtain the wet weight (*W_w_*). Subsequently they were dried in an oven at 70 °C for at least 24 h and weighted again to obtain the dry weight (*W_D_*). The water uptake was calculated according to Equation (1):

(1) $$WU\;\left(\%\right)=\frac{W_{W}-W_{D}}{W_{D}}\cdot 100\%$$

where *W_W_* and *W_D_* is the weight of wet and dry ion exchange layer.

### 2.5. Area-Specific Resistance

The area-specific resistance (*ASR*) of the membrane was measured through plane in 1 M HCl_aq_. The membrane was installed in a two-compartment test cell in a way that it separated the two compartments with an exposed area (*A*) of 19.63 cm^2^. The cell was equipped with 4 electrodes. Two auxiliary electrodes were placed at the end of each compartment (opposite to the membrane). Two reference electrodes were placed in capillaries with openings close to the membrane surface on both of its sides. Polarization (I–V) curves (galvanostatic sweeps) were recorded with current up to 1 A (current density 51 mA.cm^−2^) using a 10 mA.s^−1^ scan rate. A potentiostat (Origaflex OGF01A, Origalys SAS, Rillieux-la-Pape, France) was used to control the experiment. The electrical resistance value (*R*) was taken from the slope of the linear region the polarization curves following Ohm’s law.

Before the measurement, the membranes were equilibrated in a 1 M HCl_aq_ electrolyte solution for 24 h. The *ASR* was determined by computing the difference of the cell resistance with (*R_mem_*) and without (*R_bkg_*) the membrane and by normalizing to the active area (*A*), following Equation (2):

(2) $$ASR\left(Ω.cm^{2}\right)=\left(R_{mem}-R_{bkg}\right)\cdot A$$

### 2.6. Permeability to Vanadium Cations

The permeability of cationic species across the membrane was tested for the model case of vanadium ions (VO^2+^). The permeability cells consisted of two compartments separated by the tested membrane (*A* = 19.63 cm^2^). The donor compartment was filled with 180 mL of 0.15 M vanadyl sulphate (VOSO_4_) in 3 M H_2_SO_4_ while the receiving compartment was filled with 180 mL of 0.15 M magnesium sulphate (MgSO_4_) in 3 M H_2_SO_4_. Magnesium sulphate was used to balance the osmotic pressure on both sides of the membrane. The permeability was evaluated by monitoring the increase of VO^2+^ species in the receiving compartment resulting in a change of color of the initial receiving solution. In case of noticeable changes, the permeation of vanadium species was analyzed using a UV-Vis spectrometer.

### 2.7. VRFB Single-Cell Performance Test

A vanadium RFB cell was chosen as a convenient benchmarking system for the membrane performance. The experiments were performed in a laboratory test cell with an active membrane area of 20 cm^2^ (Pinflow Energy Storage s.r.o., Prague, Czech Republic). The membrane was mounted between two felt electrodes with dimensions of 50 mm × 40 mm × 4.6 mm (PAN GFD 4.6 EA, SGL Carbon SE, Wiesbaden Germany). The felts were activated in a furnace at 400 °C for 48 h prior to use. Commercial vanadium electrolyte (1.6 M equimolar mixture of V^3+^ and VO^2+^ ions in 2 M sulfuric acid, GfE GmbH, Nuremberg, Germany) was used. 60 mL were circulated at a flow rate of 40 mL min^−1^ in each-catholyte and anolyte-side of the cell. Nitrogen was bubbled through the anolyte during the experiment to suppress oxidation of vanadium in lower oxidation states by atmospheric oxygen. The charge and discharge of the cell was controlled using a potentiostat (Origalys SAS OGF05A–Origalys SAS, Rillieux-la-Papecity, France). The cycling test was carried out at constant current densities of: 20 mA.cm^−2^, 50 mA.cm^−2^ and 80 mA.cm^−2^ with upper and lower limits of the charge/discharge voltage set at 1.75 V and 0.80 V, respectively. Based on the charge-discharge characteristics energy, voltage and coulombic efficiencies were calculated according to Equations (3)–(5).

(3) $$CE\left(\%\right)=\frac{\int I_{d}\;dt}{\int I_{c}\;dt}\cdot 100\%$$

(4) $$EE\left(\%\right)=\frac{\int V_{d}\;I_{d}\;dt}{\int V_{c}I_{c}\;dt}\cdot 100\%$$

(5) $$VE\left(\%\right)=\frac{EE}{CE}\cdot 100\%$$

where *I_d_* and *I_c_* are the discharging and charging current, while *V_d_* and *V_c_* are discharging and charging voltages.

### 2.8. Self-Discharge Experiment

The permeation of vanadium cations across the composite membrane in an assembled VRFB cell was evaluated by monitoring the cell open-circuit voltage (OCV, self-discharge testing). The experiment was performed in the previously described single cell used for cycling experiments, but using only 10 mL of electrolyte per half-cell. The cell was charged to 1.70 V, then the open circuit voltage was monitored in time until it decreased to 0.80 V. The decrease in the OCV value, evidence of the self-discharge of the cell, is attributed to the diffusion of vanadium cations (VO_2_^+^, VO^2+^, V^2+^, V^3+^) through the membrane.

### 2.9. Chemical Stability in VO_2_^+^ Solution

The oxidation stability of the membranes was assessed in a room temperature ageing test. 1 × 2.5 cm^2^ membrane fragments were immersed in 10 mL of solution of vanadium (VO_2_^+^) in sulfuric acid at room temperature for 1000 h. The same commercial vanadium electrolyte described above was used to prepare the solution of VO_2_^+^ by charging it in the single-cell to 1.70 V and holding the voltage until the current decreased below 10 mA. After that, the catholyte was used as a source of oxidative species for testing the stability of the membranes. Potential oxidative degradation in the membrane was followed by monitoring changes in the oxidation state of vanadium in the ageing solution and detected by UV-vis spectroscopy (Thermo Scientific™ Evolution 60S UV-Visible spectrophotometer). The samples of the ageing solution were diluted ten times with ultrapure water for the measurement.

## 3. Results

### 3.1. Ion Exchange Coating Formulation and Fabrication

**Figure 1:**
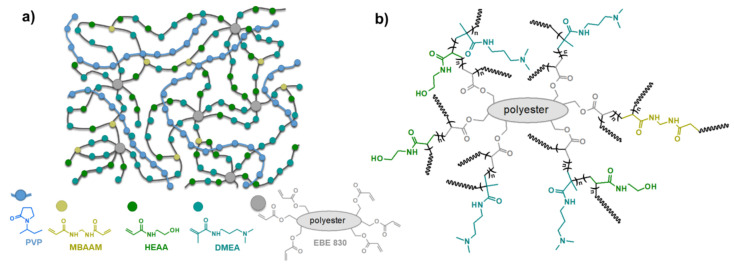
(**a**) Illustrative schematic depiction of the cured IPN of the anion exchange coating layer of the hierarchical membrane. PVP polymer chains (blue) are trapped by entanglement in the crosslinked, UV-cured matrix composed of acrylic resin (grey) and acrylamides (Yellow, green, light blue). (**b**) Depiction of a possible polymer structure of the reacted UV-cured matrix with the chemical structures of the used compounds.

Ion exchange layers were successfully fabricated on top of the porous PVC-Silica substrate. The ionomer layer adhered well to the rough surface of the PVC-Silica substrate both in dry and wet state. The water-soluble PVP was immobilized in a UV-cured polymeric matrix formulated of monomeric acrylamides and the acrylate resin. The UV cured matrix of the IPN containing PVP is crosslinked due to the use of bifunctional and hexafunctional radically reactive compounds (EBE 830, MBAAM). Figure 1 qualitatively illustrates structure of the coating layer (a) and a fragment of the possibly obtained crosslinked matrix (b). The UV cured matrix was dense enough to hold up to 16% *w*/*w* of PVP which otherwise would be soluble in water.

Membranes with greater content of PVP were not obtained as the coating dissolved or formed gel flakes in contact with water. Membranes with a PVP content below 6% *w*/*w* could not be prepared as the viscosity of the formulation became too low, and it would soak into the porous substrate before curing. The dimensional stability of the AEMs was granted by the porous substrate that served as a mechanical reinforcement. The mechanical properties such as tensile strength are in significant majority provided by the substrate and only a minor contribution of the coating layer can be observed (data available in the Supplementary Materials–Figure S2, Table S1).

**Figure 2:**
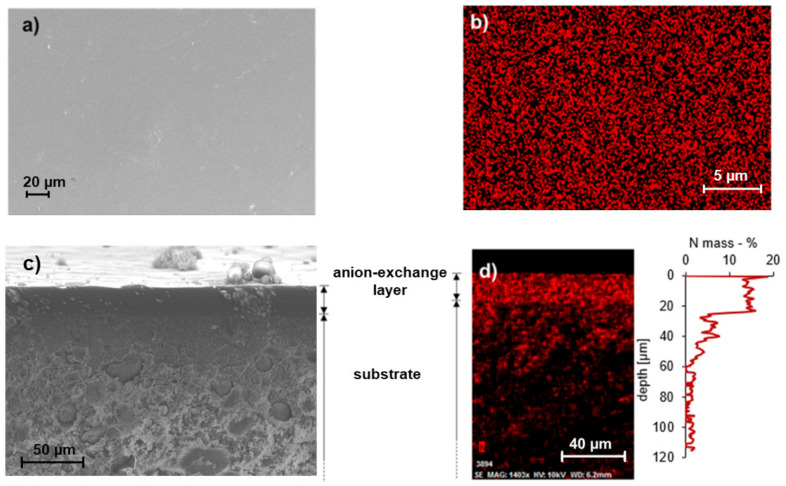
SEM/EDS micrographs presenting the structure of the composite AEM (PVP_8%). (**a**) anion exchange coating surface microscopy highlighting the coating continuity, the uniformity in the N distribution is proven by the surface EDS map (**b**), (**c**) cross section microscopy showing the hierarchical structure of the membrane: the ion exchange coating can be identified as the darker layer on top of the porous substrate, (**d**) cross section N EDS map and line scan confirming limited penetration of the anion exchange coating formulation in the porous substrate.

The membranes were examined under a SEM, as shown in Figure 2. The micrographs show that the coating is intact and smooth (no cracks and defects, no visible signs of strain). The coating was homogeneous in structure and in composition as proven by EDS data (Figure 2b). This confirms that the formulations were brought to a proper solution state for coating, and the curing process did not lead to a phase separation of the components.

Cross-sections of the membrane were also observed under a SEM (Figure 2c). The interface between the coating and substrate was clearly identifiable, indicating that the formulations had a high enough viscosity to prevent soaking into the pores of substrate before curing. EDS data (Figure 2d) show a steep step in the line scan as nitrogen is no longer detectable at 30 µm under the coating/substrate interface—this depth of penetration (35 µm) can be considered limited when compared to the thickness of the substrate (600 µm).

The average thickness of the anion exchange layer was found to be 35.4 µm with a standard deviation of 8.9 µm. The exact content of PVP in the formulation did not impact this parameter. Several exemplary cross-section micrographs used to assess the coating thickness are shown in Supplementary Materials Figure S3. The relatively high deviation in the coating thickness is attributed to the uneven surface of the substrate, originated by its fabrication process, and leading to local differences in the film’s thickness.

### 3.2. Area-Specific Resistance

**Figure 3:**
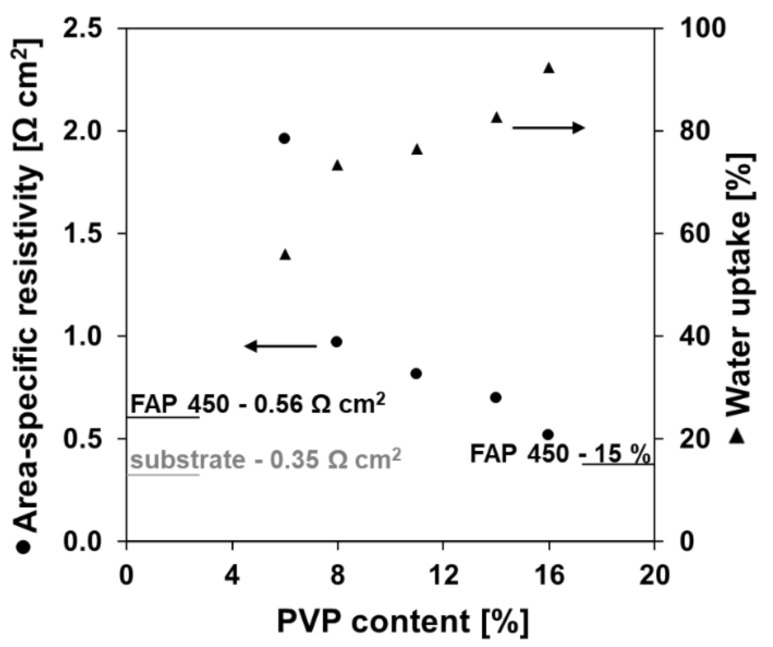
Area-specific resistance (1 M HCl, scan rate 10 mA.s^−1^) and anion exchange coating water uptake of the fabricated membranes. Values for a commercial reference (FAP 450) and the bare substate are indicated with lines in the plot.

Figure 3 reports the area-specific resistance (*ASR*) of the composite membranes as a function of the PVP content in the coating layer. In the plot, the *ASR* value for FAP 450 (black line at 0.56 Ω.cm^2^) is marked as a reference point for a commercially available anion exchange membrane. The value recorded for a bare (not coated) substrate is also reported (grey line at 0.35 Ω.cm^2^) to illustrate the effective contribution of the coating to the total membrane resistivity. A lower content of PVP in the coating layer caused a higher *ASR* of the membrane (Figure 3). A significant increase of the *ASR* can be observed when the PVP content falls below 8% (from 0.89 Ω.cm^2^ to 1.99 Ω.cm^2^), while at higher polymer contents the changes are less steep. This can be understood as at 6% PVP the water uptake of the coating is only 56% compared to 73% at 8% of PVP. At low PVP contents the densely crosslinked matrix limits the swelling of the functional coating, resulting in a greater *ASR*. The water uptake of the coating increases with the growing content of PVP, providing for easier ion transport. Membranes with 14% and higher content of PVP exhibit low *ASR*–comparable to the commercial reference FAP 450 and other commercial membranes studied by other authors [55].

### 3.3. Permeability to Vanadium Ions

**Figure 4:**
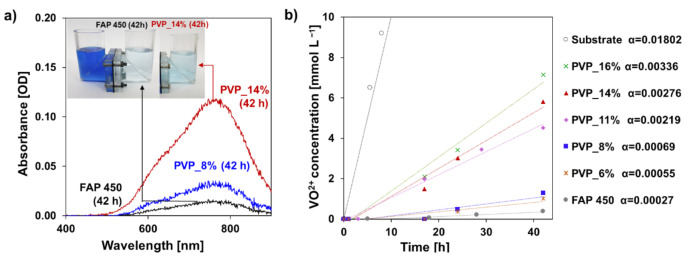
(**a**) UV-Vis spectra of receiving solutions at 42 h from the beginning of the permeability test (PVP_14%, PVP_8% and FAP 450), the picture inset shows the permeability test cells at the time of sampling; (**b**) anion exchange membranes permeability test results–VO^2+^ concentration vs. time, the α coefficients are calculated as the slopes of vanadium concentration curve in a function of time.

The composite anion exchange membranes were proven to be effective in slowing down the transport of VO^2+^ ions. Figure 4a pictures the diffusion test cells for the sample PVP_14%, PVP_8% and the commercial reference FAP 450 after 42 h from the beginning of the experiment. Permeation of vanadium ions through the membrane can be noticed by the change of color in the receiving solution, and confirmed by the UV-Vis spectra [54]. To obtain a more quantitative estimate of the diffusion rate and compare the different samples, the absorbance at 775 nm proportional to the VO^2+^ concentration in the receiving cell was measured at regular time intervals.

Figure 4b reports the results of the experiment. All the points marked for the fabricated membranes fall between the data measured for FAP 450 (grey points) (slowest diffusion–lowest slope) and the bare separator (black points) (fastest diffusion–highest slope). The transport of VO^2+^ ions was observed to be slower for membranes with a lower content of PVP. The samples containing 6% and 8% of PVP showed the slowest diffusion rates, approaching the one of FAP 450. This can be explained by higher crosslinking which leads to a lower water uptake and more steric hindrance in ion diffusion processes. Another impact to ion transport differences between the two samples may come from the fact that the amine rich matrix can become protonated under acidic condition and contribute to the coatings positive charge. In parallel, higher PVP contents lead to a gradually faster permeation of VO^2+^ ions, as a result of less densely crosslinked structure of the IPN-type coating layer thus, high water uptake.

**Figure 5:**
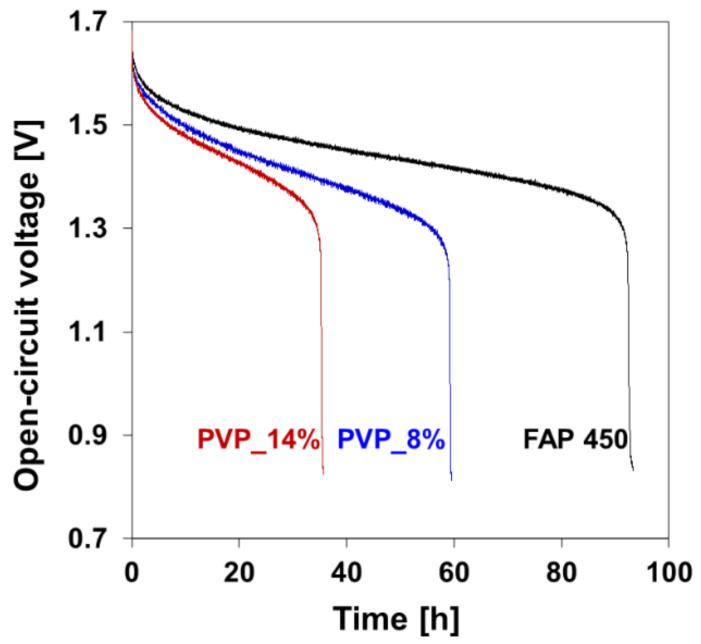
Open-circuit voltage as a function of time–self discharge curve recorded for FAP 450 and two composite membranes (PVP_14% and PVP_8%).

Self-discharge curves were recorded for the samples cycled in the RFB cell (Figure 5). As a result of its lower vanadium permeability, the sample PVP_8% allowed for a longer charge retention compared to PVP_14%. The gap in the performance of the two samples was however found to be smaller than expected from the permeability data, probably due to the different vanadium species involved in this experiment and the oxidative character of the charged electrolyte to which the membranes were exposed in this experiment.

### 3.4. VRFB Single Cell Performance

**Figure 6:**
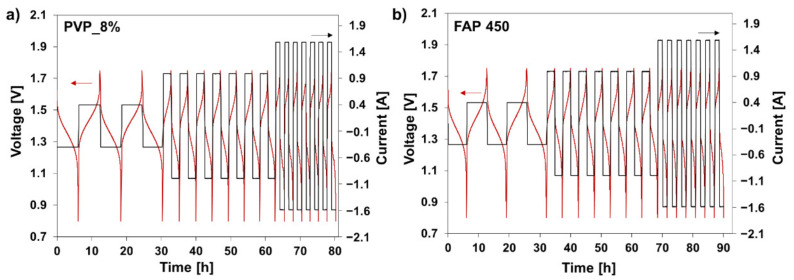
Cycling curves (90 h) of the VRFB single cell (20 cm^2^ of active area) assembled with PVP_8% (**a**) and FAP450 (**b**), cut off voltage: 0.80 and 1.75 V.

Selected membranes were tested in VRFB cycling experiments. This RFB system was selected due to vast literature available for performance comparisons [13,33,56,57]. Two membranes having an opposite combination of properties were studied: moderate ASR-low vanadium permeability (sample PVP_8%) and low ASR–moderate vanadium permeability (sample PVP_14%). Figure 6 reports the cycling curves obtained one of the composite membranes (PVP_8%) and the commercial reference. The first charge–electrolyte conditioning-was omitted because it is not relevant to further performance evaluation.

The data show that the composite membranes can be cycled in a VRFB with satisfactory results. Voltage, coulombic and energy efficiencies were computed for all the experiments. The results are showed in Figure 6 as a function of current density. Benchmark data obtained for the commercial membrane FAP 450 are reported in grey for comparison.

**Figure 7:**
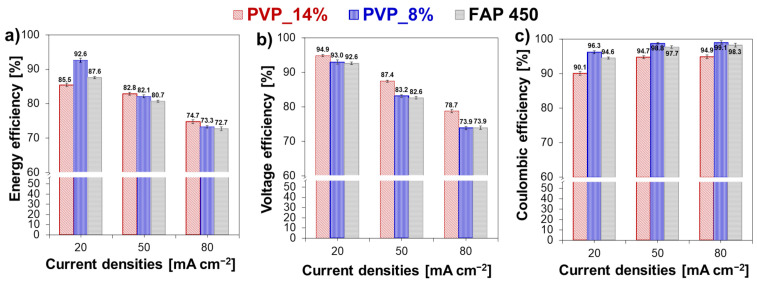
Energy (**a**), voltage (**b**) and coulombic efficiency (**c**) of the VRFB single cell assembled with FAP 450 and the fabricated hierarchical membranes prepared with 8 and 14 *w*/*w* % of PVP at different current densities.

At 20 mA.cm^−2^, the membrane PVP_8% allows to reach a higher energy efficiency than PVP_14% (89.5% in comparison to 85.5%) owing to its excellent coulombic efficiency granted by the lower cation permeability. This result could be expected from data obtained in permeability tests (Figure 4) and self-discharge experiment (Figure 5). At higher current densities −50 mA.cm^−2^ and 80 mA.cm^−2^-ohmic losses at the membrane become more significant and their effect noticeable in the voltage efficiency data (Figure 7b). The voltage efficiency becomes thus the dominant contribution in the overall energy efficiency, resulting in higher values for the more conductive PVP_14% sample (energy efficiency: 74.7% at 80 mA.cm^−2^). At these high current densities, the low ASR of the membrane compensates its lower selectivity, leading to the highest energy efficiency. Experiments at higher current densities were not performed because of the steep decay in efficiency observed already at 80 mA.cm^−2^. This is considered to be a cell limitation, present both for the proposed hierarchical membranes and the thin foil commercial membrane.

The hierarchical membranes allowed to reach energy efficiency values in line with the commercial reference. In a more detailed analysis of voltage and coulombic efficiency results, some discrepancies in comparison with the pre cycling characterization are noticeable. The FAP450 membrane showed lowest ASR and lowest vanadium permeability in the pre-cycling testing. This allowed to expect it to be the best performing membrane in cell testing throughout the entire current density range explored. In fact, the performance gap between hierarchical coated membranes and FAP 450 was not noticeable. PVP coated membranes were found to perform particularly good at higher current densities (Figure 7a). This difference in the quantitative results between pre-cycling characterization and actual cycling experiments can be pointed to the different vanadium species and the different role of electromigration phenomena in the two types of experiments.

The internal resistivity of the RFB cell was measured at the beginning of the charging step of each cycle. For the composite AEMs a minor decrease was observed through all the experiments: 64–60 Ω for PVP_14% and 82–73 Ω for PVP_8%. An opposite trend was noticed for FAP 450 (63 to 65 Ω). The decrease of internal resistivity of PVP membranes may result from degradation of the coating layer. Composite membranes after the cycling experiment did not show visible damage in SEM observation (clearly visible, continuous coating layer, Figure S5 in the Supplementary Materials).

### 3.5. Stability in Vanadium VO_2_^+^

**Figure 8:**
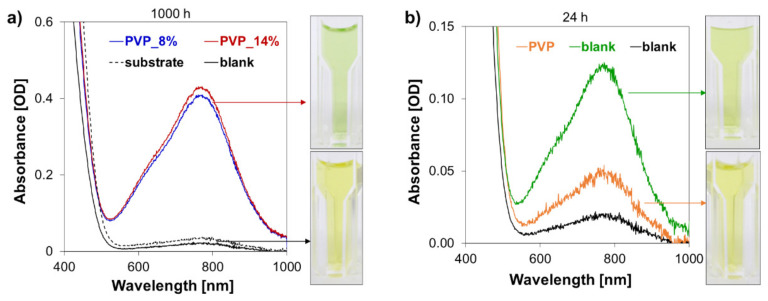
(**a**) UV-Vis spectra of the ageing solutions for the fabricated membranes after 1000 h of test, the substrate material is reported as reference. Oxidative degradation is observed for the PVP based hierarchical membranes. (**b**) UV-Vis spectra of the ageing solutions for the coating components after 24 h of test: the crosslinked matrix shows noticeably faster oxidation than PVP.

Figure 8 reports the results of the ageing test in the V_2_O_5_ (VO_2_^+^) 2 M H_2_SO_4_ solution. Diluted samples of the ageing solutions for membrane samples after 1000 h are pictured in Figure 8a. The change of color (from yellow to green), noticeable for all the hierarchical membranes samples, indicates a change in the oxidation state of the vanadium ions in the solution-and thus degradation of the ion exchange coating layers. This can be further confirmed by the appearance of an absorbance peak at 775 nm in the UV-Vis spectrum, typical of VO^2+^ species. Individual tests performed for each of the coating components (PVP and the UV cured matrix) suggest that the crosslinked aminated matrix undergoes oxidation faster than PVP (Figure 8b). Presence of hydroxide groups and tertiary amine moieties in the aliphatic chains of the polymer matrix may lead to creation of sites prone to oxidation [58,59,60].

To improve the oxidative stability of the anion exchange coatings, the tertiary amino groups present in the functional coating matrix can be quaternarized. The resulting positively charged quaternary ammonium groups are less susceptible to oxidation and more chemically stable [61]. As a simple proof of concept, the quaternarization reaction was attempted with the use of CH_3_I (procedure reported in Supplementary Materials–Section S1, Figure S4). This step has a positive impact on the membrane oxidative stability (Figure S1). The full characterization and testing of quaternarized membranes, expected to show different performance than the materials presented in this work, will be addressed in future work.

## 4. Conclusions

Anion exchange coatings based on water-soluble poly-(vinyl pyrrolidone) immobilized in an aminated crosslinked matrix have been formulated and deposited onto a porous substrate. Blade coating and UV curing were used for the fabrication process with success which encourages further exploration of use of those convenient, cost effective and environmentally friendly techniques in large scale production of ion exchange membranes. By formulating PVP together with cross linkable acrylates and acrylamides this water-soluble polymer was entrapped in an ion conductive IPN. PVP content in the material could be used for convenient tuning of the membrane ion transport properties.

The hierarchical structure of the membranes was confirmed by SEM observation and EDS analyses. Such membranes presented RFB testing performance equivalent to commercial homogeneous ion exchange membranes, resulting from comparable ASR and vanadium permeability. Properties of the membranes could be easily adjusted by changing the PVP/matrix ratio in the ion exchange coating, leading to changes in the balance between voltage and Coulombic efficiency of the RFB cell. Coatings with higher PVP content were found to have a lower ASR resulting in better VRFB performance at high current density (80 mA.cm^−2^). For the sample PVP_14%, energy efficiency values of 74.7% were calculated, higher than the value of 72.7% obtained for the commercial reference FAP 450. On the other hand, a low PVP/matrix ratio enabled a lower VO^2+^ permeability at the cost of a higher resistivity. In VRFB cycling, this resulted in higher energy efficiency at low current density (89.5% for PVP_8% vs. 87.6% for FAP450 at 20 mA.cm^−2^) while in higher current regimes this membrane showed lower energy efficiency than PVP_14%, but still a better performance than FAP 450. Moreover, the sample PVP_8% showed the highest coulombic efficiency throughout all current density range. This feature can be seen as advantageous in view of long-term application in an industrial RFB.

Ageing tests in charged vanadium electrolyte (VO_2_^+^) showed hints of oxidative degradation of the functional coating, primarily related to the crosslinked polyacrylamide matrix. A simple quaternarization modification of the coating was proposed to mitigate this issue. Apart from VRFBs the fabricated membranes can be proposed for application in other RFBs with less harsh electrolytes all-iron and organic redox flow batteries.

All things considered, the proposed approach to membrane formulation and fabrication through blade coating and UV curing a radically reactive ionomer formulation can be seen as a flexible platform allowing for a facile membrane property tuning. This is seen as an advantage in electromembrane process design, by allowing to adjust the membrane properties to specific process needs. The use of widely available precursors and simple processes in the membrane fabrication can make these materials available at a reduced cost, prevailing the development of ion exchange membrane-based energy storage technologies.

## Figures and Tables

**Table 1:** Chemical reagents, acronyms and supplier details.

Name	Acronym	Supplier
Hexafunctional polyester acrylate oligomer Ebecryl 830	EBE830	Allnex B.V.
*N*,*N*′-Methylenebis(acrylamide)	MBAAM	Merck (Sigma Aldrich GmbH)
*N*-[3-(Dimethylamino)propyl]metha-crylamide	DMEA	Merck (Sigma Aldrich GmbH)
*N*-Hydroxyethylacrylamide	HEAA	Merck (Sigma Aldrich GmbH)
Poly(vinyl pyrrolidone)	PVP	BTC Chemical Distribution GmbH
Irgacure 500 (50% 1-Hydroxy-cyclohexyl-phenyl-ketone, 50% Benzophenone)	I500	Ciba Specialty Chemicals Inc.

**Table 2:** Ratios of compounds used in the individual formulations.

	PVP [g]	DMEA [g]	HEAA [g]	MBAAM [g]	EBE830 [g]	I500 [g]
**PVP_6%**	0.85	6.3	3	0.75	2.2	0.4
**PVP_8%**	1.1	6.3	3	0.75	2.2	0.4
**PVP_11%**	1.6	6.2	3	0.75	2.2	0.4
**PVP_14%**	2.1	6.3	3	0.75	2.3	0.4
**PVP_16%**	2.5	6.3	3	0.75	2.4	0.4

## Data Availability

Data is contained within the article or supplementary material.

## Supplementary Materials

The following are available online at https://www.mdpi.com/article/10.3390/polym13152396/s1, Figure S1. SEM micrography of a typical surface of a PVC-Silica separator; Figure S2. Bone-shape samples of wetted porous separator (a) and wetted composite membrane PVP_14% (b) in a tensile strength setup; Table S1. Mechanical properties of the composite anion-exchange membrane (wet form) and uncoated porous separator (wet form): tensile strength test; Figure S3. Example of optical microscopies used for the coating thickness evaluation. Already in one membrane sample is possible to note a large deviation in the thickness membrane across the sheet; Section S1: quaternarization reaction; Figure S4. Comparison between UV-Vis spectra recorded for the quaternarized membrane and the non-quaternarized one; Figure S5. SEM image of the cross-section of PVP_14% membrane after the cycling experiments.
