# Supplementary material for: Composite Anion Exchange Membranes Fabricated by Coating and UV Crosslinking of Low-Cost Precursors Tested in a Redox Flow Battery
Source: Polymers (Basel). 2021 Jul 21;13(15):2396. doi: 10.3390/polym13152396 (PMC8347460; doi:10.3390/polym13152396)

Supplementary Material

Composite anion exchange membranes fabricated by coating and UV crosslinking of low-cost precursors tested in a redox flow battery

Martyna Charyton[1abc], Francesco Deboli[1ad], Peter Fischer[e], Gerard Henrion[b], Mathieu Etienne[c], Mateusz L. Donten[a]*

[a] *Amer-Sil S.A., 61 Rue d'Olm, L-8281 Kehlen, Luxembourg*
[b] *IJL UMR 7198 CNRS, Université de Lorraine, F-54600 Villers-lès-Nancy, France*
[c] *LCPME UMR 7564 CNRS, Université de Lorraine, 405 Rue de Vandoeuvre, 2 allée André Guinier , F-54011 Nancy, France*
[d] *Department of Chemical Engineering, KU Leuven, Celestijnenlaan 200F, B-3001, Leuven, Belgium*
[e] *Applied Electrochemistry, Fraunhofer Institute for Chemical Technology ICT, Joseph-von-Fraunhofer, Straße 7, D-76327 Pfinztal, Germany*
[1] *These authors contributed equally*
* Corresponding author: mateusz.donten@amer-sil.com

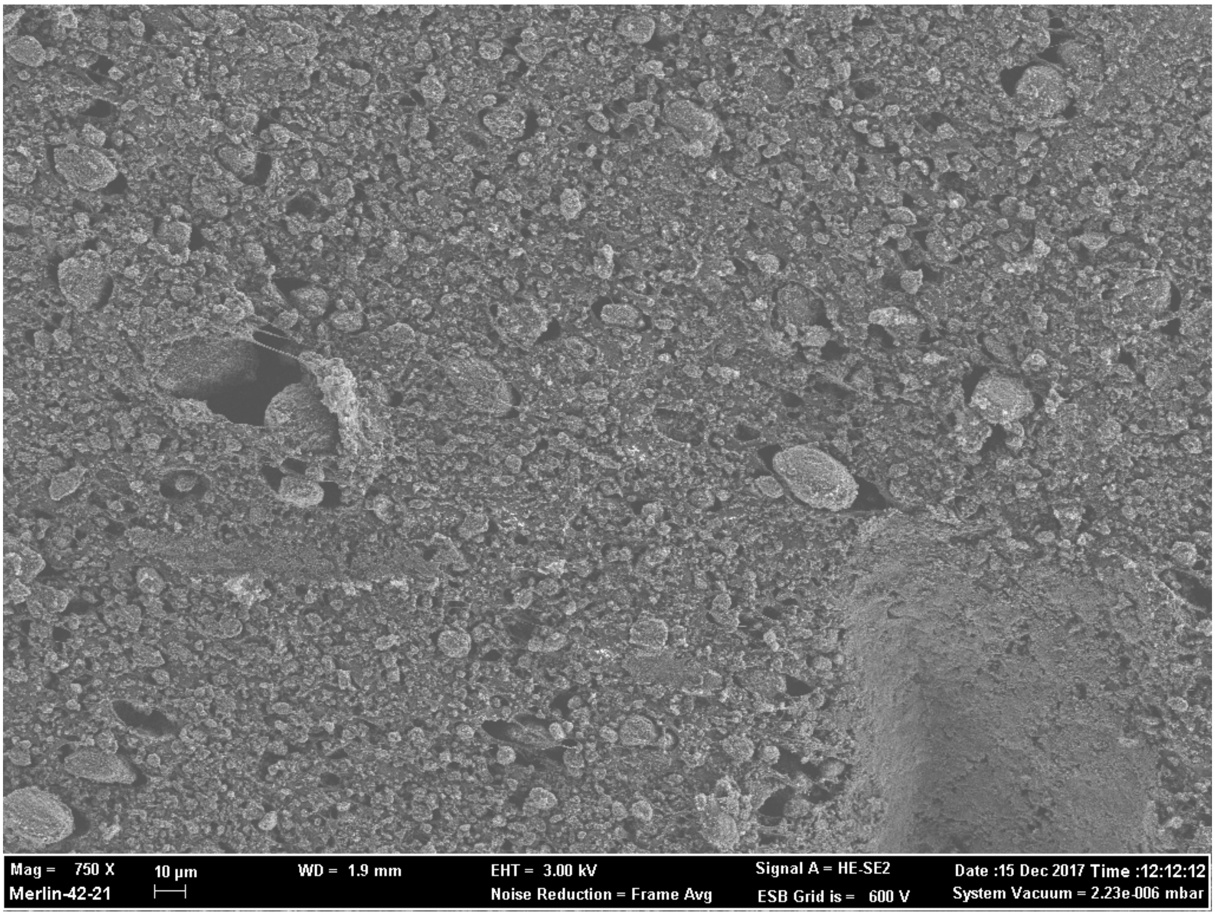

Figure S1. SEM micrography of a typical surface of a PVC-Silica separator.

Figure S2. Bone-shape samples of wetted porous separator (a) and wetted composite membrane PVP_14% (b) in a tensile strength setup.

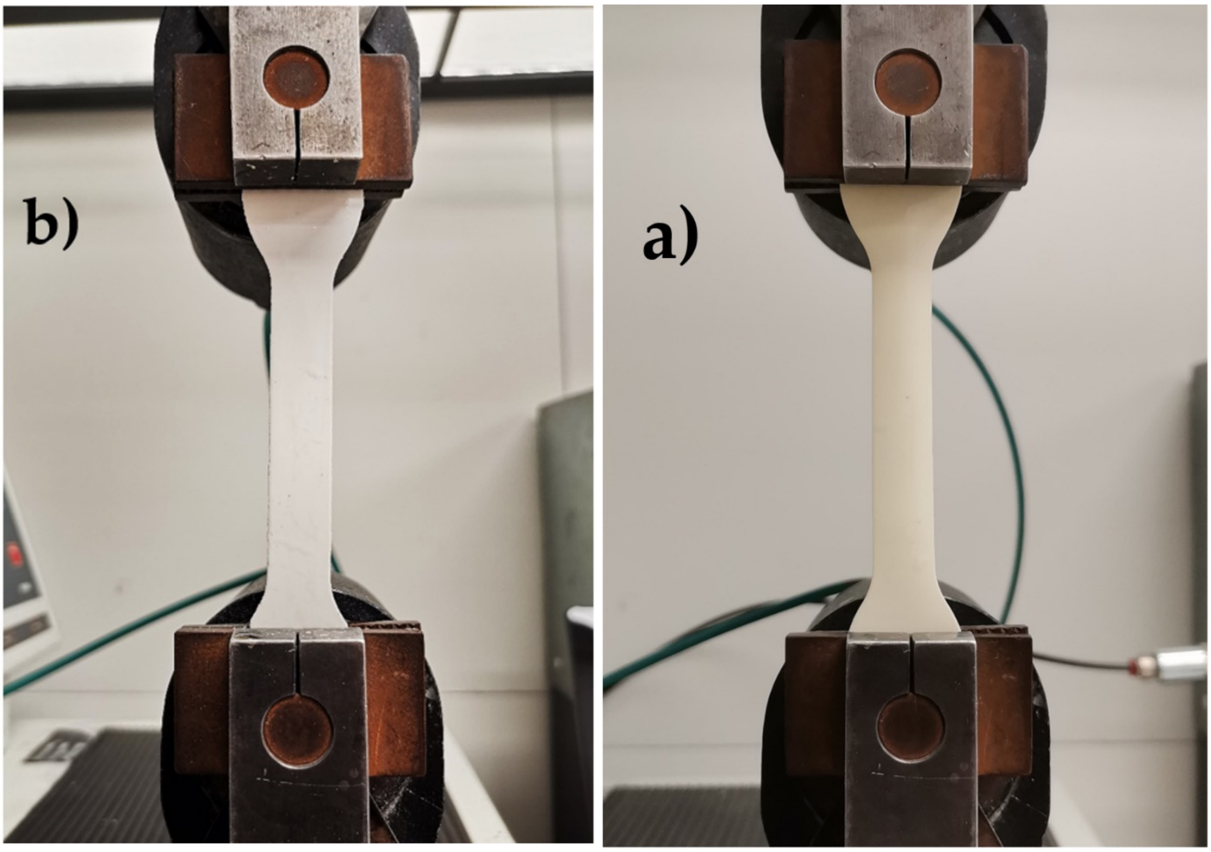

Table S1. Mechanical properties of the composite anion-exchange membrane (wet form) and uncoated porous separator (wet form): tensile strength test.

	Breaking force [N]		*Elongation at break [%]*	
	Sample 1	Sample 2	Sample 1	Sample 2
Substrate	33.15	32.67	12.66	12.11
PVP_8% (coated membrane)	35.13	34.89	11.05	11.14
PVP_14% (coated membrane)	35.96	36.34	12.31	11.62

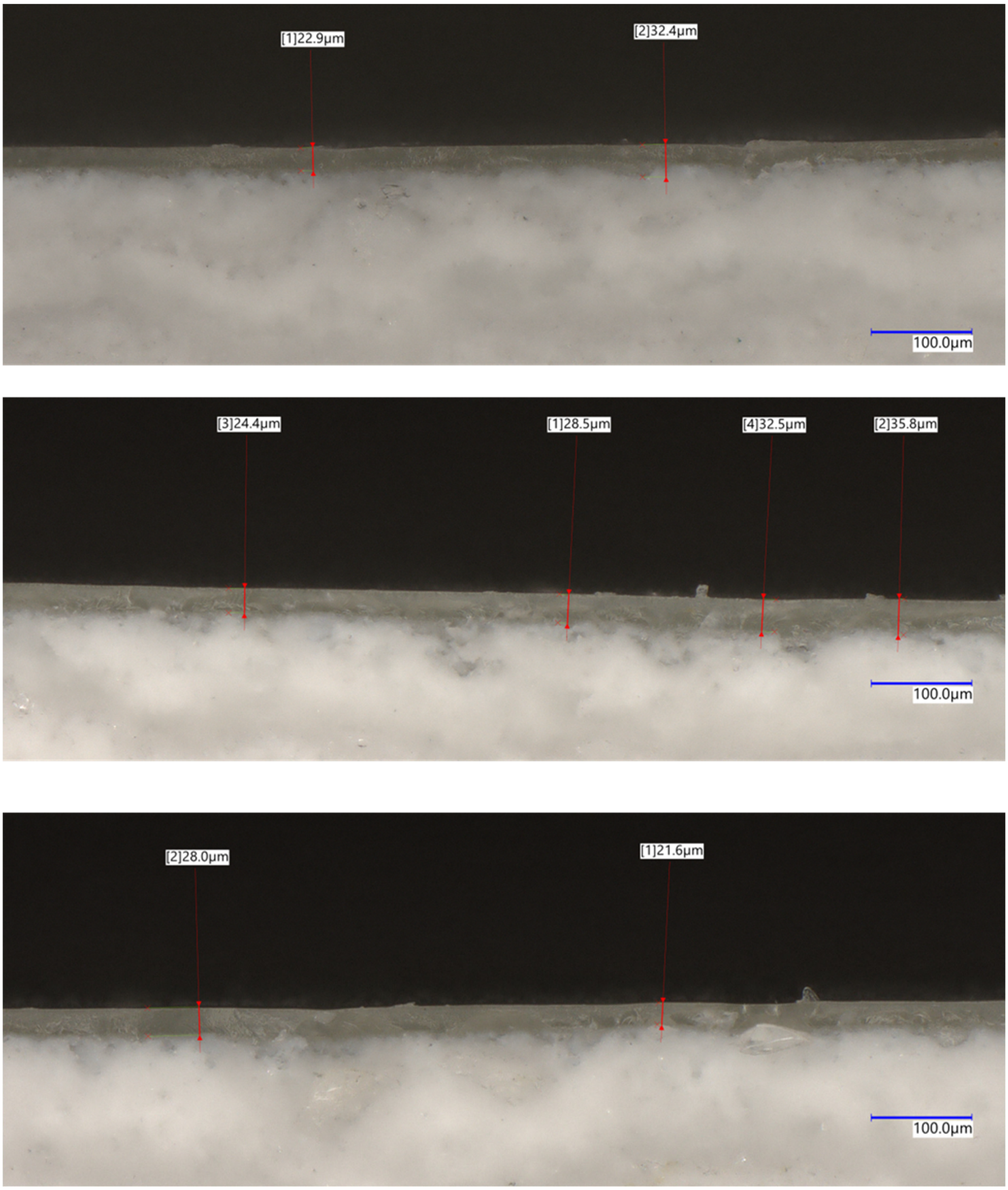

Figure S3. Example of optical microscopies used for the coating thickness evaluation. Already in one membrane sample is possible to note a large deviation in the thickness membrane across the sheet.

Section S1: quaternarization reaction

2 cm diameter samples of coated PVP_14% (known mass of substrate) were immersed in a solution of CH_3I (5%) in MeOH and mixed using magnetic stirrer for 48 h in order to allow complete alkylation of the tertiary amine groups present in the coating. After that, the membranes were rinsed with deionized water several times. In order to eliminate the excess of CH_3I used for quaternarization, the samples were left in deionized water overnight, then rinsed with deionized water. The samples were dried at 40 °C for 24 h and weighted. Then, the samples of a known coating's mass were milled and immersed in 25 ml of 0.1 M $NaNO_3$ solution to allow the exchange of counter ions. Potentiometric titration (0.02 M $AgNO_3$) was used in order to determine the equivalent point and the amount of I- in the solution. The degree of quaternarization was calculated using the following equation:

$$Nq = \frac{V_{AgNO_3} \cdot C_{AgNO_3}}{N_t \cdot 10} \, [\%]$$

V_{AgNO_3} – volume of used $AgNO_3$ to reach equivalent point [ml]

C_{AgNO_3} - concentration of $AgNO_3$ [$mol \cdot L^{-1}$]

N_t – moles of tertiary amine groups in the coating [mol]

The degree of quaternarization was indicated as 60% when used 5% CH_3I in MeOH and 20% in case of 1% CH_3I in MeOH.

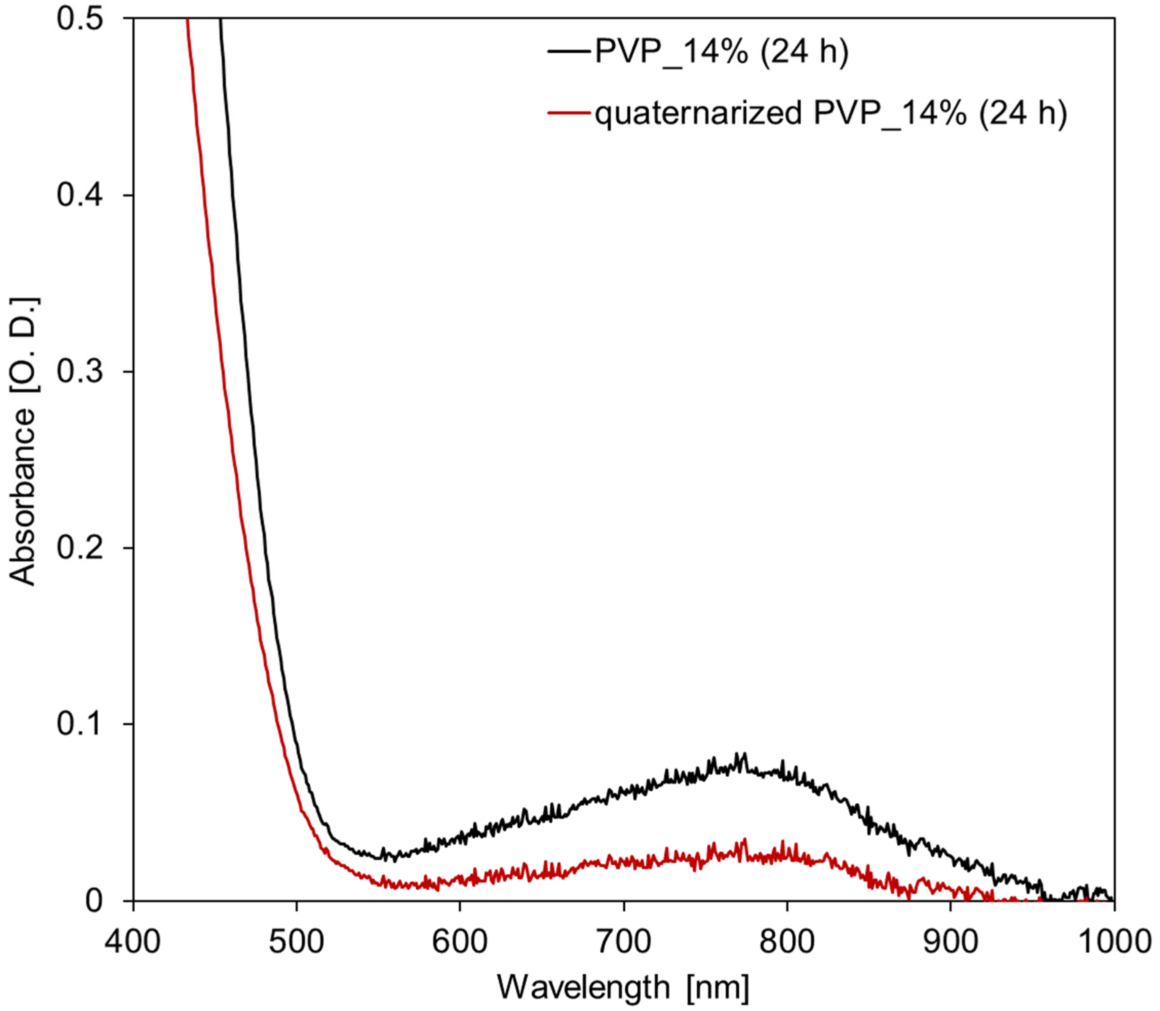

Figure S4. Comparison between UV-Vis spectra recorded for the quaternarized membrane and the non-quaternarized one.

Figure S5. SEM image of the cross-section of PVP_14% membrane after the cycling experiments.

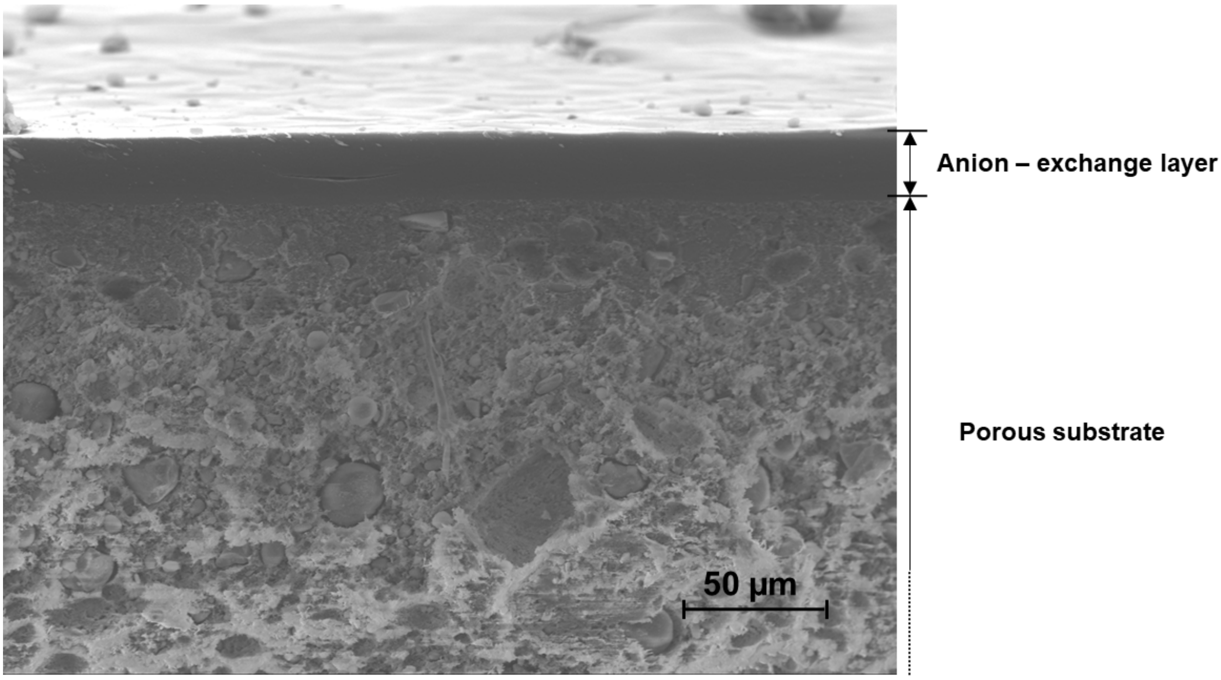

Supplement: Supplementary file 1 [file polymers-13-02396-s001.zip › polymers-1268659-supplementary.pdf]
